# Soft Arthroscopic Latarjet Procedure: Technical Note on Biceps Tendon as a Modified Sling for Restoration of Glenohumeral Stability

**DOI:** 10.1016/j.eats.2021.02.021

**Published:** 2021-05-17

**Authors:** Amr Abdel-Mordy Kandeel

**Affiliations:** Department of Orthopedics & Traumatology, Faculty of Medicine, Menoufia University, Egypt, Shebien El-kom, Menoufia Governorate, Egypt

## Abstract

As an alternate to sling glenohumeral restabilization mechanism of Latarjet procedure, recent different arthroscopic soft-tissue reconstructive techniques have been described for the management of glenohumeral instability. One of these techniques is trans-subscapularis bony tenodesis of long head of biceps (instead of coracoid graft transfer) to the anteroinferior glenoid. For simplification of the latter technique, the current article reports an alternative arthroscopic technique for management of glenohumeral instability in patients with type V SLAP lesion or poor soft-tissue quality of the anterior capsulolabral complex. In this technique, Bankart repair is followed by soft-tissue tenodesis of long head of biceps to upper border of subscapularis tendon by 2 simple stitches of non-absorbable sutures. Compared with previous ones, the currently reported technique is versatile, quick, technically simple, entirely intra-articular, and cost-saving; however, it is nonanatomic and should be investigated in biomechanical and cohort clinical studies to clarify its long-term validity.

In 2007, Lafosse et al. introduced arthroscopic Latarjet procedure to offer a minimally invasive technique for management of glenohumeral (GH) instability in patients with significant (>20%) anterior bone loss, high risk for instability recurrence (e.g., contact/competitive athletes), and revision surgery. However, it hasn’t gained much popularity among shoulder surgeons. This might be explained by its slow learning curve, long operative time, technical setup, high cost, and related complications (i.e., graft and screws malpositioning, graft resorption, and scapular dyskinesia due to pectoralis minor release). In addition, it didn’t result in significantly better outcomes in terms of functional scoring and instability recurrence.^1^, ^2^, ^3^, ^4^, ^5^, ^6^, ^7^, ^8^

Meanwhile, over the past decade, concurrent efforts were undertaken to develop novel alternatives of arthroscopic GH restabilization techniques, taking advantage of soft-tissue structures such as conjoint tendon (CT), subscapularis (SSC) tendon, or long head of biceps brachii (LHB). One of these techniques is trans-SSC bony tenodesis of LHB (instead of coracoid graft transfer) to the anteroinferior glenoid.^9^, ^10^, ^11^, ^12^, ^13^

For simplification of the latter technique, the current article reports an alternative arthroscopic technique for the management of GH instability, in which Bankart repair is followed by soft-tissue tenodesis of LHB to upper border of SSC tendon by 2 simple stitches of nonabsorbable sutures. Figure 1 demonstrates the technical principle of the reported technique.

**Fig 1:**
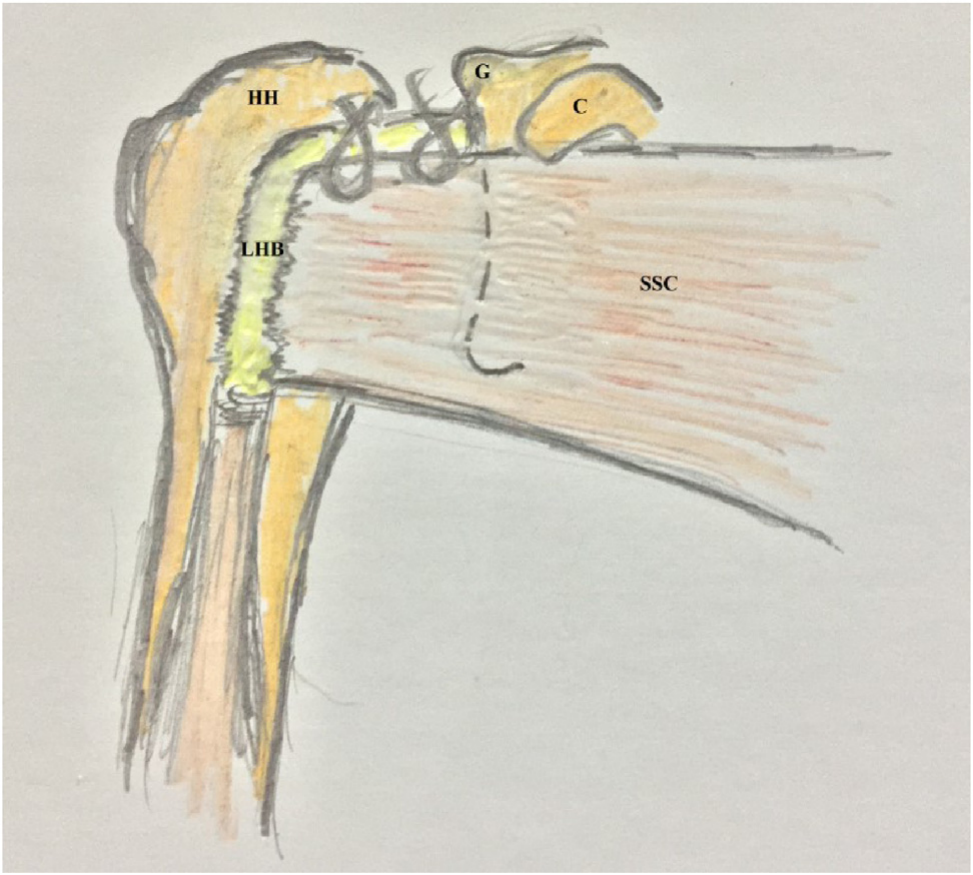
Technical principle of the reported technique (of soft-tissue tenodesis of long head of biceps brachii to upper border of subscapularis tendon using 2 simple stitches of nonabsorbable sutures during glenohumeral instability management) in the right shoulder. (C, coracoid; G, glenoid; HH, humeral head; LHB, long head of biceps brachii; SSC, subscapularis tendon.)

## Operative Technique

The present study was approved by the Institutional Committee of Scientific Research and Ethics of Faculty of Medicine, Menoufia University, Egypt. In the current article, a simplified technique is reported for the management of GH instability in patients with type V SLAP lesion or with deficient capsulolabral complex. Following general anesthesia and prophylactic antibiotic administration and with the patient in beach-chair positioning, the operated shoulder is examined for range of motion (ROM) and instability direction and grading. Then, Hill–Sachs engagement under anesthesia is assessed by placing the shoulder in 90°-90° provocative position.

Through arthroscopic standard posterior and anterior mid-glenoid portals, soft-tissue lesions are evaluated. Meanwhile, bony lesions are assessed by first calculating glenoid bone loss using a calibrated probe to exclude significant (>20%) anterior glenoid bone loss. Assessment of bony lesions also includes placement of the shoulder in 90° of abduction-45° of external rotation provocative position checking for engagement of Hill–Sachs lesion over the anterior glenoid rim.

Following exclusion of significant bony lesion necessitating bony reconstructive procedure and confirmation of type V SLAP lesion or poor soft-tissue quality of anterior capsulolabral complex, the following reported technique is performed.

### Standard Bankart Repair

Detached anterior capsulolabral complex is released from the glenoid neck using a soft-tissue liberator. Effective release is assessed by well-visualized SSC fibers and by tension-free reduction of released capsulolabral complex back to its footprint. Medially, the glenoid neck is decorticated by a motorized shaver blade to improve local biology for healing of the repaired capsulolabrum complex.

Bankart repair is accomplished through a single (i.e., anterior mid-glenoid) working portal using 3 single-loaded anchors (1.4-mm JuggerKnot All-Suture Anchors; Zimmer Biomet, Warsaw, IN) sequentially and equally spaced at 5-, 4-, and then 3-o’clock positions. A direct suture passer (Zimmer Biomet) is used to deliver both suture limbs through capsulolabral complex with adequate soft tissue between both limbs, ensuring a well-secured loop and knot of the mattress stitch.

### Soft-Tissue Biceps Tenodesis

**Fig 2:**
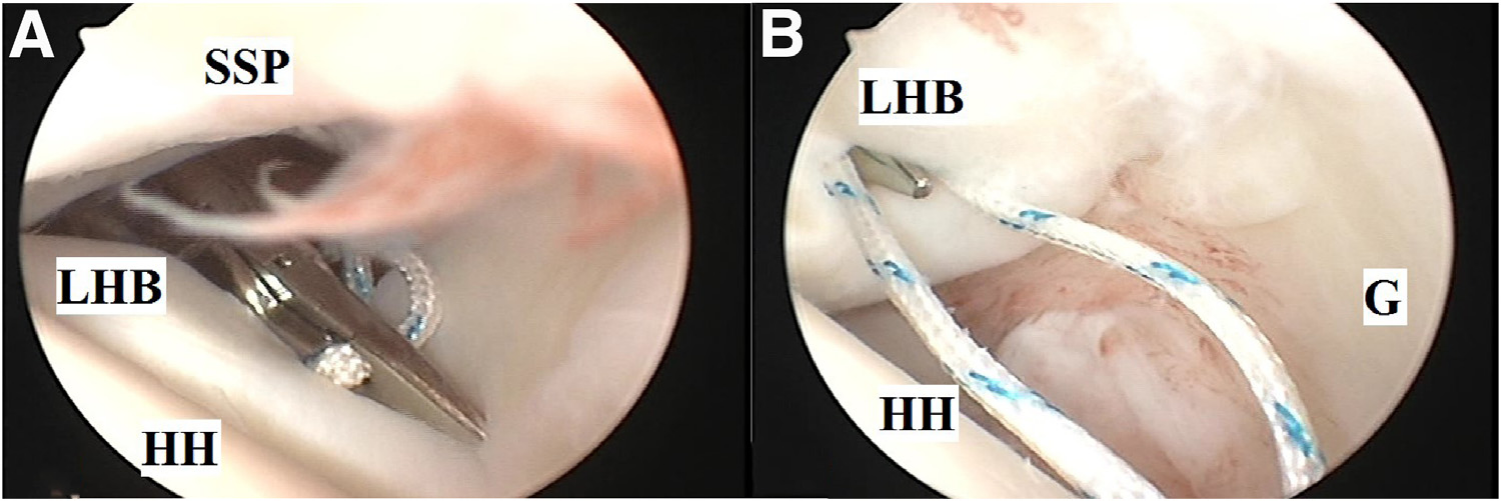
(A) and (B) #1 nonabsorbable suture loop passage through long head of biceps brachii using a direct suture passer (as a part of soft-tissue tenodesis of long head of biceps brachii to upper border of subscapularis tendon during glenohumeral instability management) in the left shoulder of a beach-chair–positioned patient while viewing from the standard posterior portal. (G, glenoid; HH, humeral head; LHB, long head of biceps brachii; SSP, supraspinatus tendon.)

Anterolateral GH portal/cannula is established 2 cm distal to the anterolateral corner of the acromion by outside-in technique using an epidural needle targeting the LHB. Through this cannula, a direct suture passer is used to pierce the LHB (just proximal to its reflection pulley) to deliver a loop of ultra-braided nonabsorbable suture (#1 MaxBraid Suture; Zimmer Biomet) through LHB. Figure 2 A and B demonstrate suture loop passage through the LHB.

**Fig 3:**
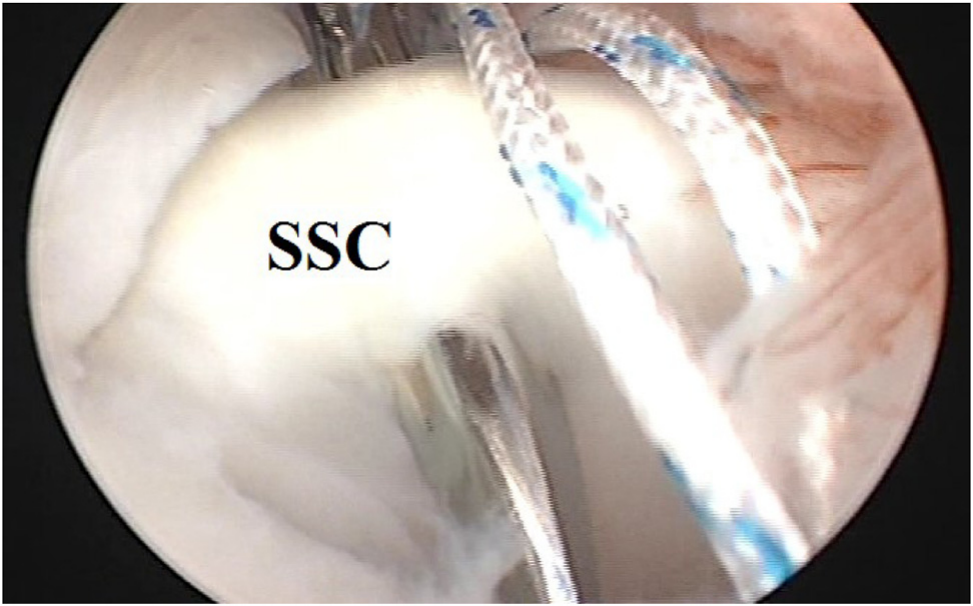
#1 nonabsorbable suture loop passage through upper border of subscapularis tendon using a direct suture passer (as a part of soft-tissue tenodesis of long head of biceps brachii to upper border of subscapularis tendon during glenohumeral instability management) in the left shoulder of a beach-chair–positioned patient while viewing from the standard posterior portal. (SSC, subscapularis tendon.)

Through the anterior mid-glenoid cannula, a direct suture passer is used to pierce the SSC tendon (at a distance of 1-1.5 cm from its superior border) near its insertion into the lesser tuberosity to retrieve the looped end of the suture through the SSC tendon to outside. Figure 3 demonstrates suture loop passage through the upper border of the SSC tendon.

**Fig 4:**
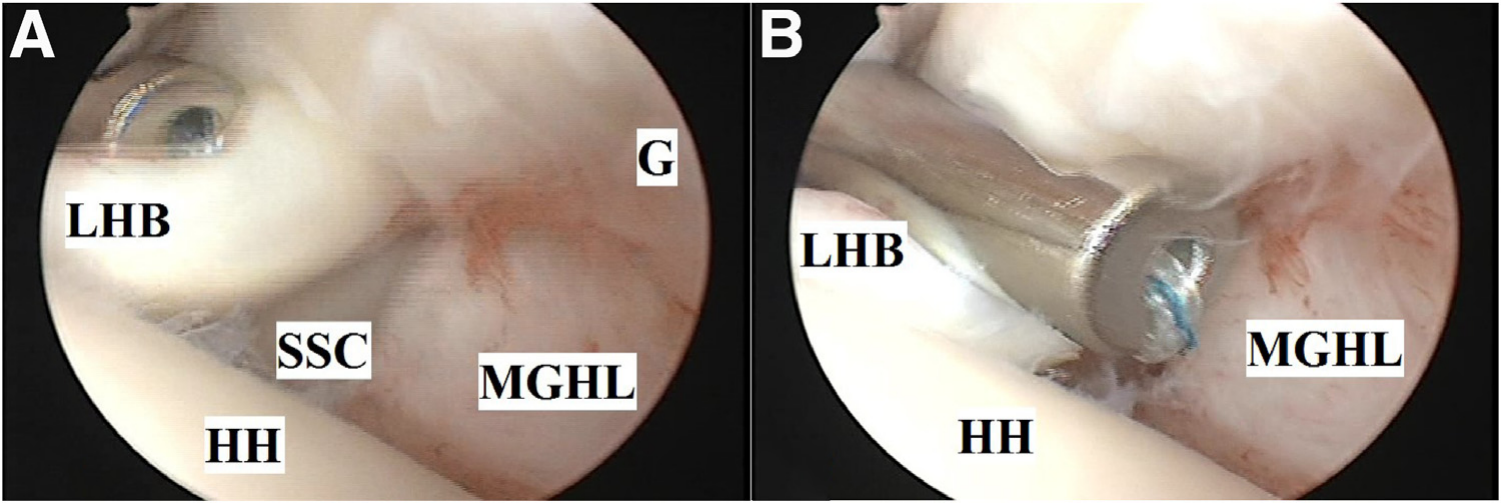
(A) and (B) Knot pusher–aided tightening of the fixed knot (to firmly hold long head of biceps brachii and subscapularis tendon to each other) and tying of alternating half hitches (as a part of soft-tissue tenodesis of long head of biceps brachii to upper border of subscapularis tendon during glenohumeral instability management) in the left shoulder of a beach-chair–positioned patient while viewing from the standard posterior portal. (G, glenoid; HH, humeral head; LHB, long head of biceps brachii; MGHL, middle glenohumeral ligament; SSC, subscapularis tendon.)

Then, a ring forceps is used to retrieve both free ends of the suture outside through anterior mid-glenoid portal. One free end of the suture is passed within the suture loop and pulled, thus driving the loop down the anterior mid-glenoid cannula till resting tightly (aided by a knot pusher) over LHB. This step ends in a fixed knot, which firmly holds the LHB and SSC tendon to each other. This knot is secured by tying free ends of the suture by 6 alternating half-hitches. Figure 4 A and B demonstrate knot pusher–aided tightening of the fixed knot and tying of alternating half hitches.

Similarly, another loop of ultra-braided nonabsorbable suture is passed within the LHB in a more proximal location preferably at midpoint between the first stitch and labral anchorage of LHB. Again, LHB is sutured to upper border of SSC tendon by employing the previous steps. Pearls and pitfalls of reported technique are summarized in Table 1.

**Table 1:** Pearls and Pitfalls of Currently Reported Technique

Pearls:
• Bankart repair is completed before LHB tenodesis • Outside-in placement of anterior and anterolateral working portals is facilitated by an epidural needle • Accurate location of LHB transfixation is essential to allow well-spaced double stitching • Accurate location of SSC transfixation is essential for secured and safe tenodesis • Technical steps should be arthroscopically well-visualized to avoid suture entanglement and loop/knot slackness • LHB tenodesis (2 stitches) should be completed before tenotomy • Integrity of Bankart repair and soft-tissue LHB tenodesis should be checked by probing before LHB tenotomy • LHB should be tenotomized very close to its labral origin to avoid second suture slippage
Pitfalls:
• Too-high SSC transfixation ends in cut-through of the suture • Too-low SSC transfixation might endanger axillary nerve • Suture entanglement might occur due to rotator interval soft-tissue interposition • Knot/loop slackness should be avoided by ensuring that the fixed knot is tightly resting (aided by a knot pusher) over LHB and is firmly holding LHB and SSC to each other

LHB, long head of biceps brachii; SSC, subscapularis.

**Fig 5:**
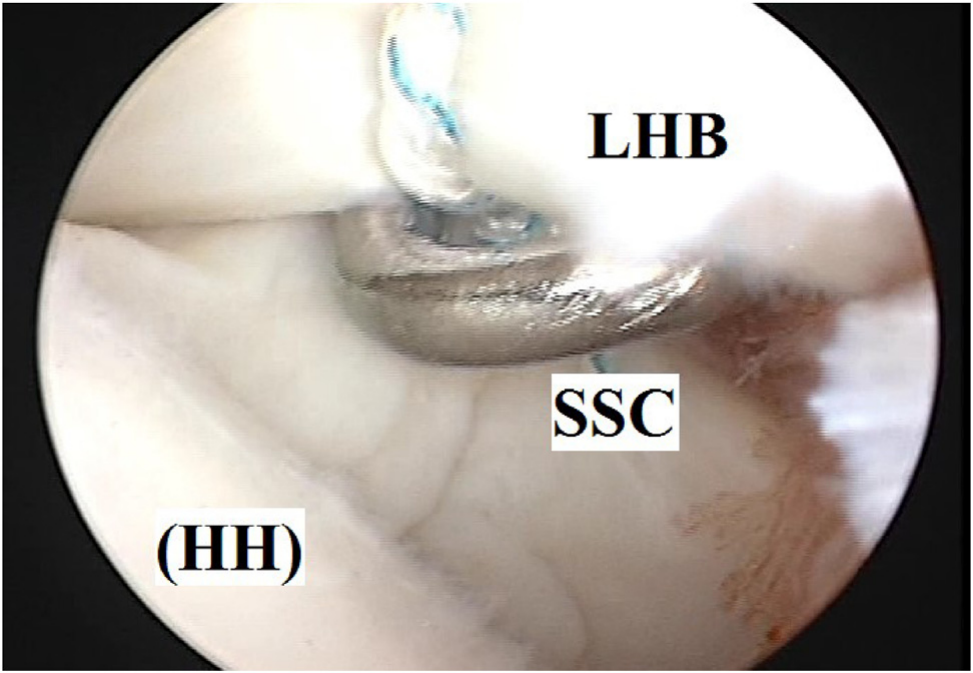
Testing (probing) of tenodesis integrity following the first stitch of soft-tissue tenodesis of long head of biceps brachii to upper border of subscapularis tendon during glenohumeral instability management in the left shoulder of a beach-chair–positioned patient while viewing from the standard posterior portal. (HH, humeral head; LHB, long head of biceps brachii; SSC, subscapularis tendon.)

**Fig 6:**
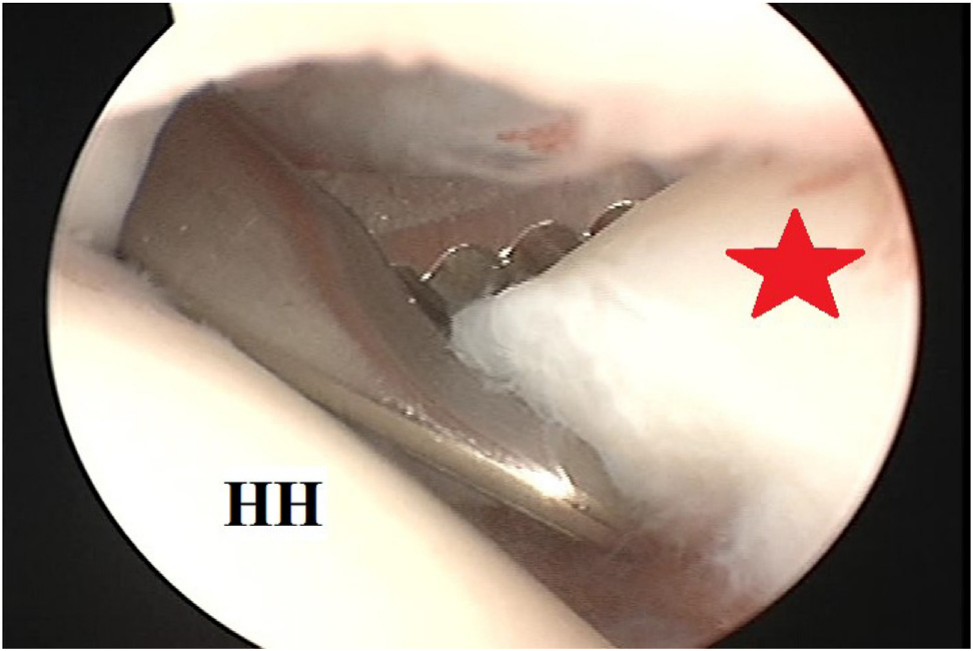
Tenotomy of long head of biceps brachii from its superior labral origin (marked as a red star) following soft-tissue tenodesis of long head of biceps brachii to upper border of subscapularis tendon (using 2 simple stitches of #1 nonabsorbable sutures) during glenohumeral instability management in the left shoulder of a beach-chair–positioned patient while viewing from the standard posterior portal. (HH, humeral head.)

Integrity of the Bankart repair and soft-tissue biceps tenodesis is checked by probing. Figure 5 demonstrates testing (probing) of tenodesis integrity following the first stitch. Then, tenodesis is completed by tenotomy of LHB off the superior labrum using a combination of diathermy probe, soft tissue biter and motorized shaver blade. Figure 6 demonstrates tenotomy of LHB from its superior labral origin.

**Fig 7:**
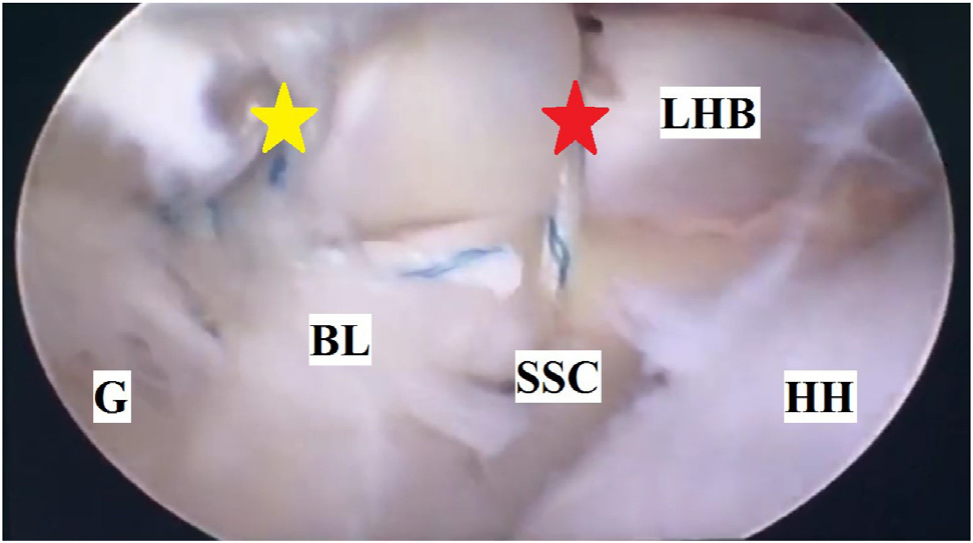
Completed soft-tissue tenodesis of long head of biceps brachii to upper border of subscapularis tendon (using 2 simple stitches of #1 nonabsorbable sutures; marked as red and yellow stars) during glenohumeral instability management in the right shoulder of a beach-chair–positioned patient while viewing from the standard posterior portal. (BL, repaired Bankart Lesion at mid-glenoid notch; G, glenoid; HH, humeral head; LHB, long head of biceps brachii; SSC, subscapularis tendon.)

Finally, integrity of the whole reconstruct is rechecked by probing before closure of the portals. Figure 7 demonstrates completed soft-tissue tenodesis of LHB to upper border of SSC tendon. For more clarification, these technical steps of the currently reported technique are illustrated in Video 1.

### Postoperative Rehabilitation

The operated shoulder is placed in broad arm sling for 6 weeks during which active (90°-150°) ROM elbow and isometric deltoid exercises are encouraged. Then, the sling is discarded, allowing light daily-living activities and practice of pendulum and assisted-active ROM exercises for 2 weeks. By 8 weeks postoperatively, the patient starts physiotherapy rehabilitation protocol of 2 weeks of passive (stretching) ROM exercises followed by 4 weeks of active (strengthening) exercises. Resisted elbow flexion and return to heavy-duty overhead and sports activities are allowed by 4 to 5 months postoperatively.

## Discussion

Lafosse et al. described the Latarjet procedure as a minimally invasive technique of coracoid transfer to the anteroinferior glenoid. However, this arthroscopic procedure is technically demanding. In practice, its clinical and radiographic superiority over its open counterpart has not yet been well-established especially with respect to bone block-related complications.^1^, ^2^, ^3^, ^4^, ^5^, ^6^, ^7^, ^8^ Accordingly, different arthroscopic nonbony techniques have evolved as alternatives to Latarjet procedure by gaining advantage of soft tissue structures as CT, SSC tendon and LHB.^9^, ^10^, ^11^, ^12^, ^13^

Considering CT, Boileau et al.^9^ reported arthroscopic Bristow “belt and suspenders” procedure involving transposition of CT through SSC split and fixation of CT within a glenoid bony tunnel by an interference screw. However postoperatively, rate of instability recurrence was still relatively high (8%) at a minimum 1-year follow-up. Taking advantage of SSC, Maiotti et al.^10^ described tenodesis of upper third of SSC tendon to anterior mid-glenoid for GH capsular deficiency. However, this technique resulted in an average loss of 6° in external rotation with the arm aside, with concerns about impaired function of upper third of SSC.

Regarding LHB, Tang and Zhao^11^ introduced arthroscopic biceps re-routing (through SSC split) and tenodesis into a glenoid tunnel created in a retrograde fashion (from posterior to anterior). Re-routed biceps was fixed within the tunnel by a suspension device on the posterior aspect of the glenoid. According to the authors, this technique can be coupled with other restabilization procedures (e.g., Bankart repair) and its indications should be limited for management of soft-tissue lesions of GH instability.

The latter technique was modified by Collin and Lädermann,^12^ who reported a similar arthroscopic tenodesis technique, however, into a 3-o’clock drilled hole in the anterior glenoid using a tenodesis screw. This tenodesis was followed by Bankart repair. Again, authors considered >30% bone loss of the anterior glenoid as a contradiction for this technique.

Recently, Milenin and Toussaint^13^ described arthroscopic trans-SSC transposition of LHB over anteroinferior glenoid using knotless anchors fort labral augmentation/reconstruction in patients with poor soft-tissue quality of the capsulolabral complex, provided that there is no significant glenoid bone loss. Nevertheless, these previous techniques are still somewhat complex, time-consuming, and costly (due to hardware).

### Indications and Contraindications

In the current article, a much more simplified technique of versatile indications is reported for patients of GH instability. Indications and contraindications of the reported technique are summarized in Table 2.

**Table 2:** Indications and Contraindications of Currently Reported Technique

Indications
Type V SLAP lesion
Poor soft-tissue quality of anteroinferior capsulolabral complex
Concomitant LHB lesions (i.e., tendinitis, <30% tear, instability, pulley lesion)
High-risk patients for recurrence of GH instability (hyperlaxity, contact/competitive sports activity)
Revision management of GH instability
Contraindications
Critically deficient (>20% bone loss) anterior glenoid
Extensive intra-articular LHB lesion (relative)

GH, glenohumeral; LHB, long head of biceps brachii.

Even in cases of GH instability with concomitant SSC tear, the reported technique is still valid following SSC repair. This validity might be supported by previous reporting of consolidated soft tissue biceps tenodesis of Fleck and Field^14^ for concurrent SSC tear and dislocated LHB.

### Technical Considerations

The currently reported technique might resemble the one Baggio et al.^15^ described for isolated biceps tendon lesion; however, technically, it differs in a number of points. An important difference is number of stitches. In current technique, tenodesis is performed by 2 stitches to ensure well-secured fixation of LHB to SSC tendon to minimize the risk of Popeye deformity in such group of young active patients. These technical differences are summarized in Table 3.

**Table 3:** Technical Differences of Currently Reported Technique From That of Baggio et al.^15^

Technical Difference	Current Technique	Baggio et al.’s Technique
Decubitus	Beach-chair	Lateral
Working portals	Anterior mid-glenoid Anterolateral	Anterior mid-glenoid
Number of stitches	2	1
Location of LHB stitches	First stitch: just proximal to reflection pulley Second stitch: midpoint between the first stitch and LHB labral origin	1.5 cm from LHB labral origin
SSC tendon	Sutured by looped end of the suture	Sutured by free end of the suture
Passage of suture free end through suture loop	Following passage of suture looped end through SSC tendon	Before passage of suture free end through SSC tendon
Tenotomy	Following tenodesis	Before tenodesis
Knot	Over LHB	Over SSC tendon

LHB, long head of biceps brachii; SSC, subscapularis.

### Biomechanical Considerations

**Fig 8:**
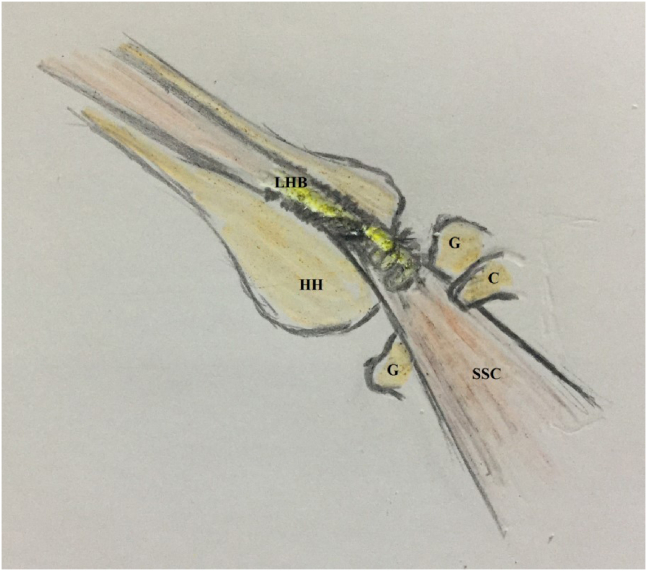
Modified sling glenohumeral restabilization mechanism of the reported technique (of soft-tissue tenodesis of long head of biceps brachii to upper border of subscapularis tendon using 2 simple stitches of nonabsorbable sutures during glenohumeral instability management) in the right shoulder. (C, coracoid; G, glenoid; HH, humeral head; LHB, long head of biceps brachii; SSC, subscapularis tendon.)

It is essential to point out that soft-tissue tenodesis of LHB to upper SSC tendon is to offer a modified dynamic sling mechanism as reported tenodesis is proximally located to site of fixation of transferred coracoid in Latarjet procedure, and additionally, LHB is smaller in cross-sectional area compared with CT. Figure 8 demonstrates modified sling glenohumeral restabilization mechanism of the reported technique.

As it is a soft-tissue procedure that doesn’t reconstruct bony glenoid, the currently reported technique is supposed to offer 3 of 4 of GH restabilizing mechanisms of the Latarjet procedure. A comparison of glenohumeral restabilization mechanisms of currently reported technique and Latarjet procedure is summarized in Table 4.^16^

**Table 4:** A Comparison of Glenohumeral Restabilization Mechanisms of Currently Reported Technique and Latarjet Procedure

Mechanism	Reported Technique	Latarjet Procedure
Sling effect	+ (LHB)	+ (CT)
Increased tension SSC fibers	+	+
Concurrent capsulolabral repair	+	+
Restoration of bony glenoid articular arc and concavity	_	+

CT, conjoint tendon; LHB, long head of biceps brachii; SSC, subscapularis.

Lacking of bony glenoid reconstruction by reported technique is not a major default, as it is described for noncritically deficient (i.e., bone loss <20%) glenoid. In addition, concavity of glenoid articular arc is re-established via concurrent Bankart repair. This might be supported by a cadaveric study of Yamamoto et al.,^16^ who concluded that bony glenoid reconstruction is not the fundamental GH restabilization mechanism of Latarjet procedure, as its maximal contribution (45%) in restoration of GH stability comes into action only during early abduction, which in turn is not the usual provocative position of GH dislocation.

Additional supportive evidence might stem from a novel biomechanical study by Mehl et al.,^17^ who concluded that currently evolving techniques of dynamic anterior shoulder stabilization by LHB tenodesis to the anterior glenoid were effective in restraining GH translation under tested instability conditions of up to 20% glenoid bone defects.

### Potential Advantages

Potential advantages of reported technique include its arthroscopic approach and versatility for different GH instability conditions as in cases of type V SLAP lesions, where it avoids increased risk of GH stiffness following anchor repair of the superior labrum. It can also be employed for patients of poor soft-tissue quality of GH capsulolabral complex, and for revision management of GH instability.

The reported technique saves time because of its simplicity and the familiarity of most shoulder surgeons with this tenodesis technique. Unlike other recent techniques, the Bankart repair is performed before biceps tenodesis to SSC while the GH joint is still capacious and has not been occupied by LHB.^11^, ^12^, ^13^

It is worth mentioning that in contrast to recently evolving techniques, the current one is an entirely intra-articular GH procedure that doesn’t violate the subacromial or retrocoracoid spaces, as it doesn’t necessitate opening of the bicipital groove, exteriorization and whip-stitching of LHB, or splitting of the SSC tendon. In addition, it is cost-saving, as it negates the use of hardware.^9^^,^^11^, ^12^, ^13^

In addition, it avoids complications related to coracoid graft (i.e., graft or hardware malpositioning, axillary or musculocutaneous nerve injury, scapular dyskinesia, and GH arthropathy). The risk of significant postoperative loss of GH external rotation is low because there is no space-occupying bone block.

Compared with the arthroscopic Latarjet procedure, revision surgery of reported technique could be relatively easier, as there is no coracoid bone block which would obstacle access to anterior glenoid.

### Technical Limitations

On the other hand, the reported technique is nonanatomic. It is expected to interfere with force-couple mechanism of 2 heads of biceps brachii reported by Itoi et al.^18^ Popeye deformity is a possibility due to failure of tenodesis. Advantages and disadvantages of reported technique are summarized in Table 5.

**Table 5:** Advantages and Disadvantages of Currently Reported Technique

Advantages:
• Sling mechanism • Versatile indications • Arthroscopic, minimally invasive procedure • Associated Bankart repair • Intra-articular tenodesis, so offering a relatively long lever arm of biceps • Technical simplicity, familiarity, quickness, safety, and reproducibility • No violation of subacromial or retrocoracoid spaces • No opening of the bicipital groove, exteriorization and whip-stitching of LHB or splitting of SSC tendon • Cost-saving (no hardware) • Avoidance of bone block and hardware-related complications • No reoperation for hardware removal • Relatively easy revision
Limitations:
• Nonanatomic • Small cross-sectional area and proximal location of LHB sling • No glenoid bony reconstruction • Technical reproducibility in extensive LHB lesions • Questionable restoration of biceps muscle-length relationship • Questionable postoperative biceps cramping pain • Possible Popeye deformity • No biomechanical validation • No long-term cohort clinical studies

LHB, long head of biceps brachii; SSC, subscapularis.

## Conclusions

For the management of GH instability in patients with type V SLAP lesion or poor soft-tissue quality of the anterior capsulolabral complex, currently reported technique of soft-tissue tenodesis of LHB to upper SSC is versatile, quick, technically simple, and cost-saving; however, this technique should be investigated in biomechanical and cohort clinical studies to clarify its long-term validity.

## Supplementary Data

Video 1 Technical steps of soft arthroscopic Latarjet procedure; soft-tissue tenodesis of long head of biceps to upper border of subscapularis tendon for the management of glenohumeral instability in left shoulder viewed from the standard posterior arthroscopic portal while the patient is seated in the beach-chair position. Following arthroscopic Bankart repair, a soft arthroscopic Latarjet procedure is performed according to the following technical steps: (1) Suture loop passage through long head of biceps brachii and then, through upper border of subscapularis tendon using a direct suture passer. (2) Retrieval of the suture loop and free suture ends through the anterior mid-glenoid cannula to outside where one free suture end is passed within the loop and pulled to drive this loop downwards along the anterior mid-glenoid cannula till resting over long head of biceps brachii and so, having a fixed knot. (3) Knot pusher–aided tightening of the fixed knot (to firmly hold long head of biceps and subscapularis tendon to each other). (4) Knot tying of alternating half hitches. (5) Another stitch is performed following the preceding steps. (6) Testing (probing) of tenodesis integrity. (7) Finally, tenotomy of long head of biceps brachii from its superior labral origin.

ICMJE author disclosure forms
